# Patient Experiences With a Mobile Self-Care Solution for Low-Complex Orthopedic Injuries: Mixed Methods Study

**DOI:** 10.2196/53074

**Published:** 2025-03-14

**Authors:** Jelle Spierings, Gijs Willinge, Marike Kokke, Sjoerd Repping, Wendela de Lange, Thijs Geerdink, Ruben van Veen, Detlef van der Velde, J Carel Goslings, Bas Twigt, Collaboration Group, N Sosef

**Affiliations:** 1 Department of Trauma Surgery, St. Antonius Ziekenhuis Utrecht, Koekoekslaan 1, Nieuwegein, 3435CM, The Netherlands, 31 634493011; 2 Department of Trauma Surgery, OLVG Hospital, Amsterdam, The Netherlands; 3 University of Amsterdam, Amsterdam University Medical Centers, Amsterdam, The Netherlands; 4 The Healthcare Innovation Center, Julius Centrum, University Medical Center Utrecht, Utrecht, The Netherlands

**Keywords:** self-care application, mHealth, experience, traumasurgery, orthopedic surgery, virtual fracture clinic, patient perspective, direct discharge, musculoskeletal injury, mobile self-care, method study, health care system, hospital, mobile health, app, smartphone, satisfactory, effectiveness, treatment, virtual clinic, virtual care, digital health

## Abstract

**Background:**

The Dutch acute health care system faces challenges with limited resources and increasing patient numbers. To reduce outpatient follow-up, direct discharge (DD) has been implemented in over 30 out of 80 Dutch hospitals. With DD, no routine follow-up appointments are scheduled after the emergency department (ED) visit for low-complex, isolated, and stable musculoskeletal injuries. This policy is supported by information leaflets, a smartphone app, and a telephone helpline with human support. Growing evidence shows that DD is satisfactory, safe, and effective in reducing secondary health care use, but thorough patient experiences are lacking.

**Objective:**

The aim of this study was to explore the experiences of patients with DD to ensure durable adoption and to improve the treatment protocol.

**Methods:**

A mixed method study was conducted parallel to the implementation of DD in 3 hospitals. Data were collected through a survey directly after the ED visit, a survey 3 months post injury, and semistructured interviews. Quantitative data were reported descriptively, and qualitative data used thematic analysis. Outcomes included the Bowen feasibility parameters: implementation, acceptance, preliminary efficacy, and demand. All patients who consented to the study face-to-face with one of the 12 low-complex musculoskeletal injuries were included in the study during the implementation period.

**Results:**

Of the 429 patients who started the primary survey, 138 patients completed both surveys. A total of 18 semistructured interviews were conducted and analyzed. Patients reported a median treatment satisfaction score of 7.8 (IQR 6.6-8.8) on a 10-point scale of DD at the ED. Information quality was experienced as good (106/138, 77%), and most preferred DD over face-to-face follow-up (79/138, 59%). Patient information demands and app use varied among patients, with a median frequency of use of 4 times (ranging from 1 to 30).

**Conclusions:**

This study shows that patients consider DD a feasible and safe alternative to traditional treatment, with a favorable perception of its acceptability, efficacy, applicability, and demand. Nevertheless, response rates were relatively low, and personal nuances and preferences must be considered when implementing DD. Clinicians and policy makers can use the insights to improve DD and work towards the integration of DD into clinical practice and future guidelines.

## Introduction

The Dutch acute health care system faces substantial challenges due to limited resources and a rising number of patients requiring in-hospital care [1,2]. To alleviate this pressure, virtual fracture clinics have been introduced for Orthopedic and Trauma surgery patients [3]. Direct discharge (DD) is the most basic part of a virtual fracture clinic, concerning solely low-complex, isolated, stable musculoskeletal injuries. With DD, patients with these injuries are discharged directly from the emergency department (ED) without routine outpatient follow-up. Patients receive a removable orthosis or sling and are given information summarized in a mobile self-care app, the Virtual Fracture Care (VFC) app.

Growing evidence shows that DD is a safe and effective alternative to “traditional” care with routine follow-up [4,5]. DD reduces secondary health care use without causing a shift to primary health care use, while patient-reported outcomes (eg, functional outcome and satisfaction scores) and adverse outcomes (eg, complications and persistent complaints) are comparable [3,4]. These results and the usefulness of this method during COVID-19 social distancing measurements have led to a rapid uptake of DD in the Netherlands for 12 frequent injuries at the ED [6]. Since the first introduction in 2019, over 25 out of the 80 Dutch hospitals have implemented DD as the standard of care, adding to over 100 virtual fracture clinics and DDs worldwide [7]. While this reorganization of care has yielded beneficial outcomes in terms of reducing resources with comparable patient outcomes, there is a lack of research on the experiences of patients and their relatives with DD.

Performing an in-depth evaluation from an end user perspective deepens the insight into the quality of care by gaining a deeper understanding of user experiences and exploring reasons for nonadoption or abandonment, ensuring sustainable adoption of DD (inter)nationally. The aim of this study was therefore to explore the experiences of patients with DD to ensure durable adoption and to improve the protocol.

## Methods

### Design

**Figure 1.**
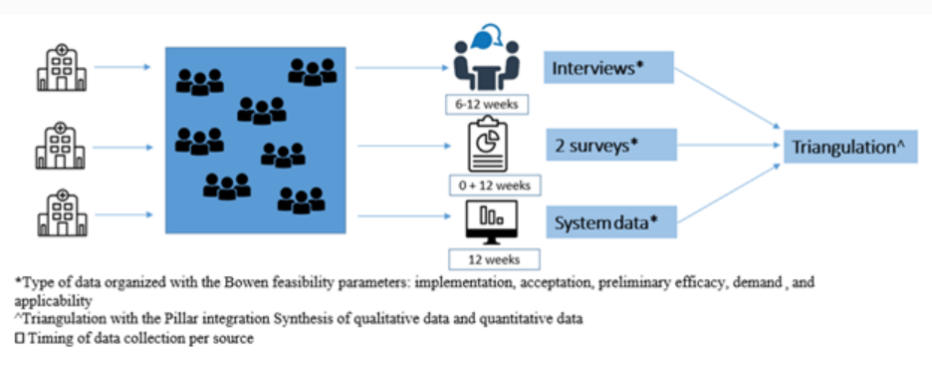
Summary of study procedures and models used to evaluate the direct discharge protocol among patients.

An observational mixed method study was conducted among patients and parents of patients younger than 12 years who sustained low-complex, isolated, stable musculoskeletal injuries parallel to the implementation of DD in 3 Dutch level-2 trauma centers from September 2021 to July 2022 with an inclusion period of 3 months per hospital (Figure 1). Quantitative data and qualitative data were collected and analyzed separately by a quantitative team (GW and JS) and a qualitative team (WL and E Mathijsen). The Bowen feasibility framework was used to organize the data within the following parameters: implementation, acceptation, preliminary efficacy, and demand [8]. After separate analyses, quantitative data and qualitative data were triangulated with the Pillar Integration Process [9]. This study was reported according to the Good Reporting on a Mixed Methods Study (GRAMMS) criteria (Multimedia Appendix 1) [10], and according to the “Improving and Standardizing Evaluation Reports of Web-based and Mobile Health Interventions from the CONSORT-EHEALTH” [11].

### Context

All participating centers were urban, level-2 trauma centers and teaching hospitals with up to 3 locations per hospital. All locations per hospital have an ED and implemented DD at the same time. Each hospital treats between 1200 and 1800 patients with low-complex, stable, isolated musculoskeletal injuries annually.

### Traditional Treatment

Before DD was implemented, patients were treated according to local trauma protocols. These protocols consisted of immobilization or support with either a cast, sling, bandage, or splint and brief information about the injury at the ED. At least 1 outpatient follow-up appointment was scheduled at the plaster room or in the outpatient clinic within 2 weeks after the injury for review, extensive information, and definitive management planning.

### Direct Discharge Protocol

This protocol was derived from the British model of a virtual fracture clinic and adapted to the Dutch health care setting in 2018. In its Dutch adaptation, DD includes 12 treatment protocols for low-complex, stable traumatic orthopedic injuries with additional injury-related criteria (Multimedia Appendix 2) [6]. Patients who met the injury-related inclusion criteria in Multimedia Appendix 2 and spoke Dutch or English fluently were included. No further predefined restrictions (eg, age or comorbidity) were used. Patients were excluded from the protocol if they had initial treatment in another hospital, follow-up in another hospital (eg, closer to home), multiple injuries, the reason for follow-up other than the injury (eg, social-care reasons), Eye/Motor/Verbal-score<15 at presentation, or intoxication. With DD, patients were discharged directly from the ED without routine outpatient follow-up. They receive a removable orthosis or a sling (eg, brace instead of a cast), and extensive information at the ED, summarized in a mobile self-care app, the VFC app. Apart from the criteria in Multimedia Appendix 2 and Dutch or English language skills (level B1), the protocols did not contain any predefined restrictions (eg, age or comorbidity). If, however, the ED staff believed physical follow-up was the most suitable treatment, outpatient follow-up was scheduled accordingly.

Patient eligibility for the protocol was re-evaluated on the next workday (within 24 h) by a team consisting of an (orthopedic) trauma surgeon and a radiologist. Patients who were incorrectly discharged directly were contacted by phone and scheduled for a face-to-face appointment.

### VFC App

**Figure 2.**
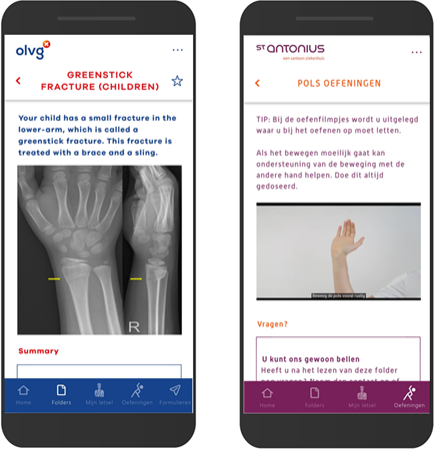
English and Dutch in-app screenshots of the Virtual Fracture Care app used in the direct discharge protocol.

The VFC app provides self-care assistance through information, videos, and a helpline and can be downloaded for free at the Google Play Store and iOS App Store (Figure 2). Injury-specific leaflets with recovery information, treatment rules, and red flags were included. Furthermore, frequently asked questions, audiovisual exercise-, immobilization-, and analgesic instructions were included to assist patients. If patients required human contact in addition to the information, a helpline operated by an employee (eg, plaster technician) was available during working hours. The VFC app aimed to increase self-management and self-care during recovery and to substitute face-to-face follow-up. No reminders were sent, and use was voluntary. The app was developed by OLVG to reduce health care use and built by medical doctors with IT experience, usability testing was performed with peers. Due to its success, it was implemented in other hospitals. No major changes occurred during the study period.

### Study Population

Patients who met inclusion criteria and consented to participate in the surveys in the VFC app were included. Based on the annual number of patients (1200‐1800 per hospital), and the inclusion time of 3 months per hospital, the estimated number of eligible patients was between 900 and 1350. Parents were asked to fill out the surveys if patients were 12 years or younger. If patients were between 13 and 16 years of age, patients and parents were allowed to complete the surveys. Patients older than 16 years could fill out the surveys alone. The exclusion criteria for this study were the same as the previously mentioned exclusion criteria for DD.

### Sampling and Recruitment

Before discharge from the ED, in a face-to-face setting, eligible patients were asked to download and open the VFC app. An in-app pop-up asked for informed consent to participate in 2 surveys and an interview. After informed consent, patients were redirected to a Research Electronic Data Capture (REDCap) environment, a web-based survey system, to fill out the primary survey and were given an opt-out form by the treating physician, [12]. Additional information was given about the study and withdrawal methods. In the REDCap environment, all participants who started the survey were invited to participate in the interview by clicking a button and providing their email addresses. Age, sex, type of injury, and hospital were used to select a purposive sample among the quantitative population for interview patients. Eligible patients were contacted a minimum of 6 weeks after injury to schedule a semistructured interview. During data collection, authors WDL and E Mathijsen considered whether the qualitative data had gained sufficient depth to perform a thorough analysis. Patients were reminded by email to complete the survey, and the second survey was sent 3 months post injury. Additional patient data were collected from electronic patient records.

### Data Collection

Data were collected from surveys, semistructured interviews, and system data.

#### Surveys

Two surveys, 1 directly after the ED visit and one 3 months after the ED visit, with 63 questions, including close-ended questions, multiple-choice questions, 5-point Likert Scales, visual analog scales, and free-text questions, measured 5 Bowen feasibility parameters (Multimedia Appendix 3) [8]. As no golden standard for the evaluation of innovations exists, the surveys and topic list were developed by 4 researchers (JS, GW, BT, and THG) and checked by 2 experts on relevance: a professor in trauma surgery (JC Goslings) and an associate professor in-process evaluations of health care innovations (JCA Trappenburg). We pretested the survey with 5 patients to improve clarity.

#### Semistructured Interviews

Two independent researchers specialized in qualitative research, not involved in daily clinical practice or the VFC research team, conducted digital semistructured interviews to minimize social-desirability bias. The interviews were held within 6 and 10 weeks post injury to warrant an optimal recall. The interviews were guided by a topic list based on literature, including previously mentioned Bowen feasibility parameters (Multimedia Appendix 4) [8]. The research team piloted the topic list for clarity and completeness and modified it during data collection.

#### System Data

Quantitative data were extracted from the electronic patient record after 3 months of follow-up. The patient characteristics included compliance to therapy, complications (yes/no), type of complications, follow-up (yes/no), type of follow-up, and imaging. Downtime from the app was extracted from the log record of the VFC app.

### Data Analysis

Quantitative data were analyzed using the SPSS (version 27; IBM Corporation) [13]. Baseline characteristics were reported descriptively using numbers and proportions for categorical variables and mean with SD or median with IQR as appropriate. The normal distribution of continuous data was assessed with visual analysis. Discrete variables were reported as numbers (percentages of the total population). The paired *t* test or the Mann-Whitney *U* test was used to determine the statistical significance of parametric variables for normally and nonnormally distributed data.

Qualitative data were analyzed according to the principles of the 6 phases of thematic analysis by Braun and Clark [14]. We have used an inductive, categorical approach because the triangulation process started after 4 phases. Data analysis started after the first 5 interviews. Interviews were audiotaped, transcribed verbatim, and analyzed using the software program NVivo (QSR International) [14]. One researcher (WL) independently analyzed the data, and another researcher (E Mathijsen) reviewed the analysis. Two researchers (WL and E Mathijsen) used several methods to ensure reliability and validity [15-17]. Discrepancies and remarks were discussed until they reached a consensus about data interpretations. Memos were made to track research decisions during analysis. Code saturation was reached when no new categories or themes emerged from the new raw data [15,16]. Instead of steps 5 and 6 according to Braun and Clark [14], we organized the data per theme during the triangulation session. The final themes were used to describe the value and feasibility of DD from the perspective of patients.

### Triangulation

After the separate quantitative and partly qualitative analyses, findings were triangulated with a simplified approach of the Pillar Integration Process technique [9]. This approach uses a transparent and rigorous 4-stage technique for integrating and presenting qualitative and quantitative findings in a joint display Microsoft Excel, 2018 [18]. One of the researchers presented the quantitative findings (JS) per study parameter, and another the qualitative findings (WDL). (Dis)similarities and self-contained themes were objectified. One of the researchers (E Mathijsen) merged these themes into a meaningful narrative (the pillar), reviewed by researchers JS and WDL.

### Ethical Considerations

This study, including the process analysis, was reviewed and approved by the Medical Ethical Committee of Utrecht, Netherlands (W21.261). Patients provided consent for participation in the research and could opt out at any time after request by email. The original consent and institutional review board approval covers secondary analysis without additional consent. A data key is stored at the local hospitals in a secured map and coded file. This is only accessible to JS and GW. The accessible data have been deidentified as far as possible (eg, age in years instead of the date of birth). Patients received no compensation to participate in this research.

## Results

### Demographics

**Figure 3.**
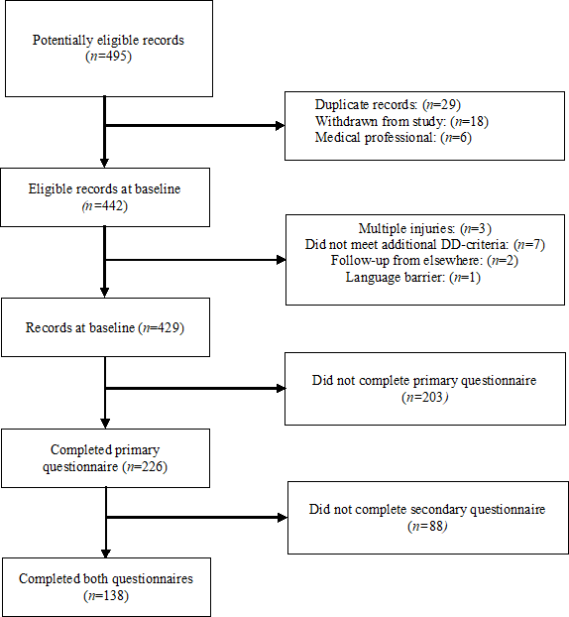
Flow diagram of eligible patients to evaluate the DD protocol. DD: direct discharge.

Of the 429 patients who started the primary survey, 203 did not provide any data or contact details and did not complete the first survey and 88 did not complete both surveys (Figure 3). Of the 138 patients that completed both surveys (response rate: 138/429, 32%), 83 out of 138 (60%) were female, and the median age was 50 years (IQR 12 to 61) (Table 1). Patients who provided contact details at baseline varied significantly from responders regarding age (*P*=.01) but not sex (*P*=.14). Most patients were native Dutch speakers, who had attended primary school in the Netherlands (136/198, 98%), and over half had a minimum of a bachelor’s degree (82/198, 59%) (Table 1). In addition, 18 patients sampled from the quantitative source participated in a web-based semistructured interview, of which 15 (83%) patients were female and 6 (33%) patients were parents of children (Table 2).

**Table 1.** Baseline characteristics of included patients in quantitative analysis of direct discharge.

Characteristic	Included in quantitative analysis (n=138)
Age (years), median (IQR)	50 (12‐61)
Age younger than 18 years, n (%)	51 (37)
Sex (female), n (%)	83 (60)
Country of primary school, n (%)
Netherlands	136 (98)
Philippines	1 (1)
Italy	1 (1)
Primary language, n (%)
Dutch	136 (98)
Filipino	1 (1)
Italian	1 (1)
Highest level of education, n (%)
Elementary school	4 (3)
High school	21 (15)
Vocational school	28 (20)
College	50 (36)
University	32 (23)
Other	3 (2)
Hospital treated, n (%)
Center A	54 (39)
Center B	26 (19)
Center C	58 (42)

**Table 2.** Demographics of interviewed patients or parents regarding experiences with the direct discharge protocol.

ID	Sex	Type of injuries	Year of birth
#1	Female	Mallet finger	1969
#2	Female	Weber A Fx^a^ or avulsion Fx	1971
#3	Female	Weber A Fx or avulsion Fx	2000
#4	Male	Fifth metatarsal Fx	1956
#5	Female	Radial head or neck Fx	1957
#6	Female	Ankle distortion	1992
#7	Female	Radial head or neck Fx	1963
#8	Female	Torus Fx of the radius	2011
#9	Male	Radial head or neck Fx	1959
#10	Female	Hallux Fx	1964
#11	Male	Fifth metatarsal Fx	1980
#12	Female	Hallux Fx	2010
#13	Female	Spoke injury	2017
#14	Female	Greenstick Fx	2014
#15	Female	Radial head or neck Fx	1974
#16	Female	Torus Fx	2014
#17	Female	Torus Fx	2011
#18	Female	Weber A Fx or avulsion Fx	1956

a Fx: fracture.

### Implementation

Both data sources indicated that most patients were satisfied with their ED visit and the introduction of the app, despite some mentioning the hectic ED environment and difficulty downloading the app due to poor Wi-Fi connection. The physiological distress, and the hectic ED environment, resulted in an inability to recollect all the information provided by the ED physician. Several patients pointed out that the information in the VFC app was a valuable supplement to their ED visit (quote 1). Mobile app stability was excellent, with only 1 patient reporting issues accessing the app. The helpline could not be reached in 4 cases, resulting in 2 patients contacting the ED directly. The app was available 99.6% of the time, with the only downtime (5 hours in 3 months) caused by a software problem that was fixed by the app builder.

*Because… it always goes quickly. It is always busy. But still, you know, this was completely clear; what I could expect and that I could download the app.*

[quote 1; ID #10]

### Acceptation

The median satisfaction with treatment was 7.8 (IQR 6.6-8.8) on a 10-point scale. Qualitative data complemented quantitative data, as most patients mentioned that they were satisfied with DD (quotes 2 and 3).

*I would give DD a 7 or 8. Yeah, let’s say 7, because I do feel that it might be difficult for older people. Especially because they don’t always understand technology, you know.*

[quote 2; ID #15]

*To be honest, I think it’s better. You know, often it’s like, you go to a hospital, and it takes half a day just to get there, and come back, and all those things, and then it’s just like: “O, it looks fine.”*

[quote 3; ID #5]

### VFC App Acceptation

Most patients (106/138, 77%) reported that the quality of the information in the app was good and reported that the information answered the questions they had during their recovery (82/138, 58%). Additionally, 59% (79/138) would prefer the app over face-to-face follow-up if they were to have a similar injury. However, in qualitative data, some patients expressed concerns that the app may not be suitable for people with limited digital skills. Even though none of the patients identified themselves as “vulnerable,” many reported that the app may not be appropriate for vulnerable individuals. Patients expressed that DD should not be mandatory for all in order to protect potentially vulnerable patients for whom DD would not be suitable.

### Brace Acceptation

A total of 122 patients out of 138 (89%) used the brace for the prescribed period. Most patients (116/138, 84%) removed the brace or the sling during showering, at night (118/138, 86%), and (72/138, 52%) during non–weight bearing exercises. Most patients (97/138, 71%) found the brace comfortable, and 94 out of 138 patients (68%) found the brace convenient for these types of injuries (Multimedia Appendix 5). These findings were complementary to the qualitative data. Patients preferred the brace to a cast as it allowed for better mobility because it was less bulky and less rigid compared with a cast (quote 4). However, the increased mobility made it tempting to overexert oneself, resulting in increased pain and insecurity for some patients (quote 5). The smaller size of the braces made some injuries seem less serious and burdensome, reportedly leading to social pressure to return to work earlier than advised by the doctor or app. Additionally, the less rigid nature of the brace resulted in increased skin friction, causing a superficial ulcer in 1 patient in the qualitative data.

*I really like the brace because it allowed me to move. So, I was not stuck with a bulky cast on my leg, but rather, I had a lot of flexibility. I could actually determine what I wanted to do or not. So, I found that very enjoyable.*

[quote 4; ID #2]

*The brace was sometimes the reason I went over my limit. I could move more and was in the process of moving to a new home. But then the pain came back. I found it very difficult to ‘guard’ my limits.*

[quote 5; ID #18]

### Helpline and Perceived Safety

Most patients found the helpline important (116/138, 84%) and that the helpline offered them a sense of security (86/138, 62%). For some patients, the helpline was a way of checking if their recovery was still on track (quote 6). Most patients (94/138, 68%) in both data sources expressed that treatment with a brace and a helpline is safe for these types of injuries. However, some expected that if injuries were more severe, they would need more assistance than a brace and a smartphone app (quote 7).

### Self-Empowerment

An increase in perceived self-empowerment was reported in 67 out of 138 (49%) of all patients, 51 (37%) patients reported neutral results, and 20 (14%) patients reported no increase. Enhanced treatment engagement was reported in 58 (42%) patients, while 50 (36%) patients reported neutral results, and 31 out of 138 (23%) patients reported no enhanced engagement (Multimedia Appendix 6). In the qualitative data, some of the interviewed patients stated that this type of treatment provided more self-empowerment and therefore, more control in their recovery (quote 8).

*After about a week or three, I had a setback. I couldn’t find this in the app, so I decided to call the helpline. The pain came back, and I was afraid I had broken something or something like that. They reassured me that it could not happen so quickly and that I just needed to rest for 24 hours h. They were right! It was nice to be able to check this.*

[quote 6; ID #11]

*Yes, I believe it is safe, given the circumstances. Because it wasn’t that serious. Yes. I do have the confidence that if it is something serious I won’t receive this type of treatment.*

[quote 7; ID #17]

*It was nice that I could read what I was allowed to do and what I was not. I think that gave me more control over my recovery. I knew what I could do myself in terms of exercises, and that was very helpful.*

[quote 8; ID #2]

### Preliminary Efficacy

#### Secondary Health Care Use

A total of 10 out of 138 (7%) patients had a face-to-face follow-up, and 9 (7%) patients by phone. Two patients attended the ED again after discharge due to anxiety and pain at the fracture site and were scheduled for outpatient follow-up. Two patients received follow-up for a wound check and 2 received follow-up as decided by the medical specialist due to severe pain complaints at the ED.

#### Functional Outcome

More than half of the patients had fully recovered 3 months after injury in terms of daily activities, sports, and work (84/138, 61%). Between 31% and 40% of patients were limited in functional outcomes once or twice a week. Approximately one in 10 patients were limited in physical activities more than 3 times a week. (Multimedia Appendix 7).

#### Pain

Most (92/138, 67%) patients have used painkillers in the first 3 weeks of recovery. Of these patients, 70 out of 92 (76%) patients have used acetaminophen, 17 (12%) patients have used nonsteroidal anti-inflammatory drugs, and 5 (4%) patients have used other undefined analgesics. Few patients (7/92, 5%) used cooling of the injury site to reduce pain. Analgesic use in the second week was lower (35/92, 38%) and further declined in the third week (9/92, 10%) and in the fourth week or after (9/92, 10%). Qualitative data showed limited pain complaints after immobilization of the initial trauma. Pain complaints in the first weeks were treated with analgesics. After the immobilization, a few patients had persistent pain symptoms. Most of these patients sustained a Weber A ankle fracture or ankle distortion. Those patients were most limited in their daily activities, specifically during more intense physical activities (eg, labor and sports) (quote 9).

*The eight weeks of recovery are over, I believe. So now I should be able to start exercising again, well I do karate but I’ll wait a little longer for that.*

[quote 9; ID #4]

#### Demand

Almost all patients used the app during recovery (133/138, 95%) with a median of 4 times (IQR 2-6.5, range 0‐30). Reasons to use the app included checking recovery exercises (91/138, 68%), treatment rules (69/133, 51%), the phase of recovery (68/133, 51%), seeking helpline information (57/133, 42%), and analgesics (10/133, 8%). Qualitative data supported the quantitative data. App use was focused on the first weeks post injury, and parents consulted the app more than children. They occasionally showed it to their child, predominantly if the information contained photos and videos (quote 10). Among the interviewed patients, almost all patients reported not requiring face-to-face follow-up for these types of injuries. The biggest advantage of DD is that it is time-saving on a personal level. Nevertheless, a few patients expressed a preference for outpatient follow-up. Especially if they had persistent complaints or when the recovery was slower than expected. Patients expressed that this led to insecurity, which was also expressed by patients who used the app more frequently or later in the recovery phase (quote 11).

A minority of patients reported a lack of human contact and the physicians’ reassurance of adequate recovery. Subsequently, as a minimal substitute, these patients suggested a feedback system (eg, pain scores or communication tools) to assist them further.

*As for the app, he did see it, but you know, whatever! The only thing they find really interesting are pictures. What I liked that in the app is that they actually showed what was going on and what it looks like.*

[quote 10; ID #17]

*I was constantly looking for confirmation in the app, online, or at the fracture helpline.*

[quote 11; ID #11]

## Discussion

### Principal Findings

This study shows that patients consider DD a feasible and safe alternative to traditional treatment, with a favorable perception of its acceptability, efficacy, applicability, and demand. Nevertheless, response rates were relatively low, and personal nuances and preferences must be considered when implementing DD.

### Comparison With Literature

Patient satisfaction and perceived safety with DD aligned with previous studies [3,19,20]. Most patients responded positively to the introduction of DD at the ED, brace treatment, and assistance with the VFC app and helpline. The VFC app proved valuable in overcoming low recall of verbal information due to the chaotic ED environment and psychological distress. This finding is consistent with a systematic review highlighting the benefits of additional visual and written information in enhancing recall and injury knowledge [21]. Adequate understanding of the injury is crucial for properly following self-care protocols and monitoring red flags, this aligns with the finding that almost all patients used the app at least once. The braces used with DD were well-received, offering advantages such as easy removal (eg, during or at night), and improved daily living activities due to the early weight bearing. Braces seem to affect patients positively, as the lack of these advantages has been reported as most burdensome during cast immobilization [22]. However, the perceived decreased severity of the injury may pose a risk of overexertion and require further research.

Preliminary efficacy results align with previous research, demonstrating low complication rates, secondary health care use, comparable functional outcomes, and patient satisfaction with treatment [4,5,23]. Most patients preferred the VFC app over face-to-face follow-up for these injuries. While using apps in orthopedic and trauma surgery is not new, adding a self-care app to DD and virtual fracture clinics is a novel approach [24-26]. Patients found the information quality good and appreciated the time-saving component (eg, reducing work absenteeism). However, in the case of more severe injuries with wounds or complex fractures, some patients would prefer face-to-face interaction for additional reassurance. As previously suggested, digital self-care could be combined with face-to-face follow-up or used for preappointment education in patients with more severe injuries [27,28].

Although a good fit for most, some (young) patients noted that the app might not be suitable for older individuals or those with limited digital skills, potentially increasing health inequities in an already digitally oriented world [29,30]. However, it is important to note that DD targets relatively young patients, who are considered capable by the treating physician.

While previous studies have highlighted the potential for increased self-empowerment and patient engagement with eHealth, this study did not explicitly confirm it [31-33]. Despite positive study results, individual nuances in patients and injury types require a continuous assessment to ensure personalized patient care.

Information demand and app use varied among patients, implying different levels of demand among users. A minority of patients expressed a desire for more human contact and reassurance. A possible suggested solution was developing an in-app numerical feedback system, such as a questionnaire or communication tool.

### Strengths and Limitations

This study has several strengths. To our knowledge, it is the first to provide in-depth interviews with patients treated using DD, providing valuable insights. Second, the research team’s multidisciplinary involvement ensured a comprehensive evaluation and analysis of the data from multiple perspectives. Thirdly, the mixed method approach, including the separate collection of both data sources until data saturation, combined with the triangulation, increased the likelihood of realistic and rigorous results. Additionally, using a validated framework provided a structured insight into each feasibility parameter.

However, limitations include potential responder and selection bias due to a younger sample of patients with higher education levels and Dutch literacy rates than the general Dutch population. This age difference could be caused by the 12 selected injuries, of which half only occur in pediatric patients. Furthermore, the response rate of this study was 32%. Response rate, literacy, and education may limit the generalizability of the findings to a broader Dutch population, especially those with lower health literacy or digital skills. Furthermore, the timing to measure functional outcomes might have been suboptimal in these patients, as for some injuries (eg, mallet finger injuries), the immobilization period had just ended, resulting in limitations in daily activities.

### Implications for Clinicians and Policy Makers, and Future Perspectives

DD has emerged as a promising approach to reduce outpatient follow-up while maintaining positive effects on primary health care use, patient satisfaction, complications, and functional outcomes [4,5]. By delivering follow-up care close to home, associated health care costs and societal costs decrease [34]. In addition, it limits unnecessary patient travel to the hospital thereby reducing the environmental impact of health care. Including patients’ perspectives in evaluating new care pathways, whether digitally assisted or not, is crucial for sustainable adoption and quality of care. Clinicians, researchers, and policy makers should prioritize patient involvement throughout the design, prototype, pilot, and evaluation phases. “Design thinking,” a validated approach widely recognized in user experience and implementation literature, can be used to design these pathways. For example, replacing hospital care with DD involves changes in location and care deliverers, which presents new needs, challenges, and opportunities suitable to solve with design thinking. This study has identified areas for improvement of DD in terms of functions and features, and adjusted language to lower literacy. Future studies should focus on co-designing in-app feedback systems that address patient and health care professional needs for reassurance and monitoring like communication tools or questionnaires.

### Conclusion

Patients consider DD a feasible and safe alternative to traditional treatment, with a favorable perception of its acceptability, efficacy, applicability, and demand. Nevertheless, response rates were relatively low, and personal nuances and preferences must be considered when implementing DD. Clinicians and policy makers can use the insights to improve DD and work towards the integration of DD into clinical practice and future guidelines.

## Supplementary material

10.2196/53074 Multimedia Appendix 1 Good Reporting on a Mixed Methods Study (GRAMMS) criteria for the study: “Patient Experiences With a Mobile Self-care Solution for Low-complex Orthopedic Injuries: Mixed Methods study”.

10.2196/53074 Multimedia Appendix 2 Additional criteria and immobilization for treatment of low-complex, traumatic orthopedic injuries with the direct discharge protocol.

10.2196/53074 Multimedia Appendix 3 Surveys used to evaluate direct discharge among health care professionals.

10.2196/53074 Multimedia Appendix 4 Topic list for health care professionals the direct discharge protocol.

10.2196/53074 Multimedia Appendix 5 5-point Likert scale distribution of acceptance-related outcomes regarding experiences with a brace.

10.2196/53074 Multimedia Appendix 6 Self-empowerment and perceived safety of care with direct discharge.

10.2196/53074 Multimedia Appendix 7 Frequency of limitation per week in physical function, activities of daily living, and school or work in patients treated with direct discharge at 3 months follow-up.

## Abbreviations

DD: direct discharge
ED: emergency department
GRAMMS: Good Reporting on a Mixed Methods Study
REDCap: Research Electronic Data Capture
VFC: Virtual Fracture Care
