# Correction: Patient Experiences With a Mobile Self-Care Solution for Low-Complex Orthopedic Injuries: Mixed Methods Study

**DOI:** 10.2196/75812

**Published:** 2025-04-29

**Authors:** Jelle Spierings, Gijs Willinge, Marike Kokke, Sjoerd Repping, Wendela de Lange, Thijs Geerdink, Ruben van Veen, Detlef van der Velde, J Carel Goslings, Bas Twigt, Collaboration Group, N Sosef

**Affiliations:** 1 Department of Trauma Surgery, St. Antonius Ziekenhuis Utrecht, Koekoekslaan 1, Nieuwegein, 3435CM, The Netherlands, 31 634493011; 2 Department of Trauma Surgery, OLVG Hospital, Amsterdam, The Netherlands; 3 University of Amsterdam, Amsterdam University Medical Centers, Amsterdam, The Netherlands; 4 The Healthcare Innovation Center, Julius Centrum, University Medical Center Utrecht, Utrecht, The Netherlands

In “Patient Experiences With a Mobile Self-Care Solution for Low-Complex Orthopedic Injuries: Mixed Methods Study” (JMIR Hum Factors 2025;12:e53074) the authors noted one error.

The ninth author’s name previously appeared as:

Carel Goslings

This has been changed to

J Carel Goslings

The correction will appear in the online version of the paper on the JMIR Publications website, together with the publication of this correction notice. Because this was made after submission to PubMed, PubMed Central, and other full-text repositories, the corrected article has also been resubmitted to those repositories.

