# Supplementary material for: Patient Experiences With a Mobile Self-Care Solution for Low-Complex Orthopedic Injuries: Mixed Methods Study
Source: JMIR Hum Factors. 2025 Mar 14;12:e53074. doi: 10.2196/53074 (PMC11927796; doi:10.2196/53074)
Supplement: Multimedia Appendix 2 [file humanfactors-v12-e53074-s002.docx]

**Multimedia Appendix 2.** Additional criteria and immobilization for treatment of low-complex, traumatic orthopedic injuries with the Direct Discharge protocol

| **Injury** | **Pediatric/adult** | **Criteria** | **Immobilization after DD** |
| --- | --- | --- | --- |
| Pediatric clavicle Fx | Pediatric | Age ≤ 14 years  No indication for surgical treatment | Sling |
| Radial head-/neck Fx | Adult | Head: Mason type 1, neck: undisplaced, or  Positive fatpad sign | Pressure bandage, sling |
| Greenstick or torus/buckle type Fx of the distal forearm | Pediatric | Acceptable angulation based residual growth  Torus/buckle type: isolated ulna Fx, isolated radius Fx or both  Greenstick type: isolated ulna Fx or isolated radius Fx | Removable wrist brace |
| Fifth metacarpal neck Fx | Adult | Volar angulation < 70°  No rotational deviation | Buddy strap and pressure bandage |
| Mallet finger | Adult | Either bony or tendinous  Treated conservatively | Mallet splint |
| Weber A type ankle Fx | Adult | Dislocation < 2 mm  No signs of stage 2 supination-adduction type injury | Tubigrip and ankle brace |
| Avulsion type ankle Fx | Adult | Either lateral or medial malleolus or tarsal bones | Tubigrip and ankle brace |
| Fx of fifth metatarsal base | Adult | Fx located in either Jones zone 1 or zone 2  Dislocation ≤ 4 mm | Walker boot |
| Fx of greater toe | Both | Either proximal or distal phalanx Fx  Undisplaced | Spica pressure bandage and bandage shoe |
| Fx of lesser toe | Both | Any isolated Fx  No indication for surgical treatment | Buddy strap |

Fx: fracture

° angle in degrees

DD Direct Discharge
