# Supplementary material for: Patient Experiences With a Mobile Self-Care Solution for Low-Complex Orthopedic Injuries: Mixed Methods Study
Source: JMIR Hum Factors. 2025 Mar 14;12:e53074. doi: 10.2196/53074 (PMC11927796; doi:10.2196/53074)
Supplement: Multimedia Appendix 3 [file humanfactors-v12-e53074-s003.docx]

**Multimedia Appendix 3.** Surveys used to evaluate Direct Discharge among healthcare professionals

*Page 1*

# Baseline characteristics FEASSIDD study

Thank you for being willing to participate in the research on Direct Discharge and the Virtual Fracture Care app.

On the next page, you will be asked a few short questions, which will take a maximum of one minute. One of the required pieces of information is your email address, where the questionnaire will be sent to.

1. In which hospital were you treated?

Het Spaarne Gasthuis

Het Slingeland Ziekenhuis

Het Gelre Ziekenhuis

2. To which e-mail address can we send the survey?

__________________________________

3. Are you filling out this survey for your child

(<16 years old)?

Yes

No

4. What is your date of birth?

__________________________________

5. What is you sex?

Male

Female

4.* What is the date of birth of your child?

__________________________________

5.*What is the born sex of your child?

Boy

Girl

# Survey 1 Direct Discharge

Thank you for agreeing to participate in this brief questionnaire. This questionnaire is about your expectations and preferences regarding the treatment.

This questionnaire is part of a feasibility study on the implementation of eHealth and Virtual Fracture Care in the Netherlands. The data you provide here will be used for scientific research. By completing the questionnaire, you give consent for the anonymized use of this data for publication purposes. Additionally, you will receive a form to withdraw your participation if you no longer wish to continue.

This questionnaire consists of two parts and takes five minutes each time.

You will now receive a questionnaire and another one in three months. Furthermore, if you grant permission, you can participate in an interview. More information can be found in the letter you will receive at the Emergency Department.

6. What injury did you have?

Pediatric collar bone fracture

Radial head or neck fracture

Greenstick fracture

Torus fracture

Fifth metatarsal bone fracture

Mallet finger

Weber A or avulsion fracture of the

Minimal spoke wheel injury

Sprained ankle

Big toe fracture

Broken toe

Fifth metacarpal bone fracture

7. In three monts, we will conduct additional short digital interviews. May we approach you fort his?

Yes

No

8. What type of information did you chose?

Paper leaflet

Virtual Fracture Care app

I did not chose

9. In which country did you go to primary school?

__________________________________

10. Is Dutch your native language?

Ja

Nee

11. What is your native language?

__________________________________

*Page 3*

12. What is the highest level of education you completed?

Primary school

VMBO

HAVO

VWO

MBO

HBO

WO

Other

13. Other, being …

__________________________________

U have completed 25% of the survey.

# Experiences at the Emergency department

We would like to hear your experiences at the Emergency Department.

**Can you indicate to what extent you agree with the following statements?**

|  | Totally disagree | Disagree | Neutral | Agree | Totally agree |
| --- | --- | --- | --- | --- | --- |
| 14. I received the necessary help. | O | O | O | O | O |
| 15. The healthcare provider was polite to me. | O | O | O | O | O |
| 16. The healthcare provider listened attentively to me. | O | O | O | O | O |
| 17. The healthcare provider had enough time for me. | O | O | O | O | O |
| 18. The healthcare provider had enough time for me. | O | O | O | O | O |
| 19. The healthcare provider took me seriously. | O | O | O | O | O |
| 20. I have confidence in the expertise of the healthcare provider. | O | O | O | O | O |
| 21. The healthcare provider told me what I wanted to know about my health complaints. | O | O | O | O | O |
| 22. The healthcare provider explained things in an understandable way. | O | O | O | O | O |
| 23. The healthcare provider provided me with general information about the brace and/or sling. | O | O | O | O | O |
| 24. The healthcare provider gave me complete information about the next steps in my treatment. | O | O | O | O | O |
| 25. The healthcare provider told me when I could resume everyday activities, such as walking. | O | O | O | O | O |
| 26. The healthcare provider told me who to contact if I became concerned about my health issues after leaving the Emergency Department | O | O | O | O | O |
| 27. I received the care I expected. | O | O | O | O | O |
| 28. I feel well assisted by the care provided by the healthcare provider | O | O | O | O | O |

Thank you for your participation, you will receive the second survey in three months!

# Second survey Direct Discharge

Welcome back,

Three months ago, you visited the Emergency Department where you were treated for a fracture or bruise. With the help of this questionnaire, we would like to know about your experience with the recovery process and/or subsequent visits, as well as the quality of care provided.

29.)

Have you used the Virtual Fracture Care app after downloading?

Yes

No

30)

Why did you (not) use the application?

31) How satisfied are you with the treatment you received? (0 meaning

Very dissatisfied - 100 meaning very satisfied

tevreden)

0

50

100

*(*

*Place a mark on the scale above)*

32)

Can you (or your child) move the body part just as well as before the injury?

I can do the same as before.

I can use the limb well.

I can use the limb quite well

I can barely use it.

33)

Did you (or your child) use any analgesics after the injury?

Yes

No

34)

What kind of analgesics?

Cooling

Paracetamol

Ibuprofen

Naproxen

Other

35)

In which week(s) after injury have you used analgesics?

In the first week

In the second week

In the third week

in the fourth week

After four weeks

**Recovery (on average over the last two weeks)**

No (0 keer per

week)

Not often (1-2

per week)

Often (3-5

per week)

Very often (every day)

36)

Are you (or your child) limited in your daily functioning

37)

Are you (or your child) limited in sports activities

38)

Are you (or your child) limited in school or work?

39) How often did you use the VFC app during your recovery?

__________________________________

*Page 9*

1. I have used the application to... (multiple answers possible)
   - Obtain information about the helpline
   - View exercsies for the recovery of my fracture or bruise
   - Look up the phone number for the helpline
   - Search for information about pain relief
   - Search for information about living rules for my fracture or bruise
   - Obtain information about my phase of the treatment process
   - I did not use the application

**App quality: To which extend do you agree with the following statements?**

|  | Totally disagree | Disagree | Neutral | Agree | Totally agree |
| --- | --- | --- | --- | --- | --- |
| 1. The information in the application was of good quality | O | O | O | O | O |
| 1. I received the type of information I wanted in the application | O | O | O | O | O |
| 1. The application answered my healthcare questions during the recovery process. | O | O | O | O | O |
| 1. I would recommend this application to a friend/family member who needs similar help. | O | O | O | O | O |
| 1. I am satisfied with the advice I received through the application. | O | O | O | O | O |
| 1. The application helped me effectively manage my health complaints. | O | O | O | O | O |
| 1. Overall, I am satisfied with the application. | O | O | O | O | O |
| 1. I would use the application again if I had a similar injury. | O | O | O | O | O |

| 1. Do you have any suggestions for improvement of the app? | __________________________________ |
| --- | --- |
| You have finished over 50% of this survey |  |

# Treatment experiences regarding the VFC app: selfcare

Can you indicate to which extent you agree with the following statements?

## Treatment experiences

|  | Totally disagree | | Disagree | Neutral | | | Agree | Totally agree | |
| --- | --- | --- | --- | --- | --- | --- | --- | --- | --- |
| 1. The application gives me more control over my own recovery. | O | | O | O | | | O | O | |
| 1. The application makes me feel more involved in my treatment. | O | O | | | O | O | | | O |
| 1. The application gives me more control over my own recovery. | O | | O | O | | | O | O | |
| 1. The application makes me feel more involved in my treatment. | O | | O | O | | | O | O | |
| 1. I consider this application a good alternative to a hospital visit. | O | | O | O | | | O | O | |
| 1. I find it important to have access to a helpline (the Fracture Line) that I can rely on. | O | | O | O | | | O | O | |
| 1. The helpline provides me with a sense of safety. | O | | O | O | | | O | O | |
| 1. I perceive the treatment with a brace and the application as safe. | O | | O | O | | | O | O | |

1. Do you have any further remarks?

__________________________________

# Immobilization

61)

I have removed the brace during personal hygiene

Never

Once or twice

Often

Always

62)

I removed the brace to do exercises

Never

oefenen

A few times

Often

Always

63)

I am satisfied with the brace or sling

Totally disagree

Disagree

Neutral

Agree

Totally agree

Thank you for you participation!

No

Yes

59)

The brace or sling are comfortable

Totally disagree

Disagree

Neutral

Agree

Totally agree

60)

I have used the sling or brace for the prescribed period
