# Supplementary material for: Patient Experiences With a Mobile Self-Care Solution for Low-Complex Orthopedic Injuries: Mixed Methods Study
Source: JMIR Hum Factors. 2025 Mar 14;12:e53074. doi: 10.2196/53074 (PMC11927796; doi:10.2196/53074)
Supplement: Multimedia Appendix 4 [file humanfactors-v12-e53074-s004.docx]

**Multimedia Appendix 4.** Topic list for healthcare professionals the Direct Discharge protocol

| General questions |
| --- |
| - You visited the emergency room a few weeks ago. Can you briefly tell me how the visit went? - (opening question)   - *How did you feel when you went home?*   - *How were the first days at home?*   - *Did you experience any pain?*   - *Was the brace supportive? (if applicable)*   - *Was the information provided in the first week sufficient?* |
| Acceptation (satisfaction) |
| - What was important to you during your visit to the emergency room? In other words, what did you want and expect to be done?   - *Can you explain this further? Can you provide an example?* - To what extend did what was done align with what you wanted and expected?   - *What aligned?*   - *What did not allign? Why you think that is? What is your opinion on this?* - How satisfied are you with the care provided?   - *Can you express that on a scale of 1 to 10*   - *What prevents it from being a (-1 point) X ?* - How satsified are you with the VFC-app?   - *Can you express that on a scale of 1 to 10*   - *What prevents it from being a (-1 point) X ?* - How satisfied are you with the information provided in the app?   - *Can you explain this further? Can you provide an example?* |
| Demand / implementation |
| - To what extent was it complicated to use the VFC app?   - *Can you explain this further? Can you provide an example?* - What did you consider the biggest advantages of the VFC app?   - *Can you explain this further? Can you provide an example?* - What did you consider the biggest disadvantages of the VFC app?   - *Can you explain this further? Can you provide an example?* - How often did you use the VFC app?   - *On which occasions did you use it?* - Did you have contact with healthcare professionals through the helpline?   - *In what situation was that?*   - *What advice did you receive?*   - *To which extent did that help you?* - Did you experience any technical issues while using the VFC app? - *Can you explain this further? Can you provide an example?* - *Do you think improvements are possible, and if so, what improvements?* - If you had to go to the emergency room again with a new injury, would you be willing to use the VFC app again?   - *What makes you say that?* |
| Preliminary Efficacy |
| - What do you think about the VFC app being offered as an alternative to standard care in the hospital   - *Can you explain this further? Can you provide an example?* - To what extent did using the VFC app help you in your treatment and recovery?   - *Can you explain this further? Can you provide an example?* - To what extent did you feel safe with the digital care pathway and the use of the VFC app?   - *Can you explain this further? Can you provide an example?* |
