# Supplementary material for: Patient Experiences With a Mobile Self-Care Solution for Low-Complex Orthopedic Injuries: Mixed Methods Study
Source: JMIR Hum Factors. 2025 Mar 14;12:e53074. doi: 10.2196/53074 (PMC11927796; doi:10.2196/53074)
Supplement: Multimedia Appendix 6 [file humanfactors-v12-e53074-s006.docx]

**Multimedia Appendix 6.** Self-empowerment and perceived safety of care with DD


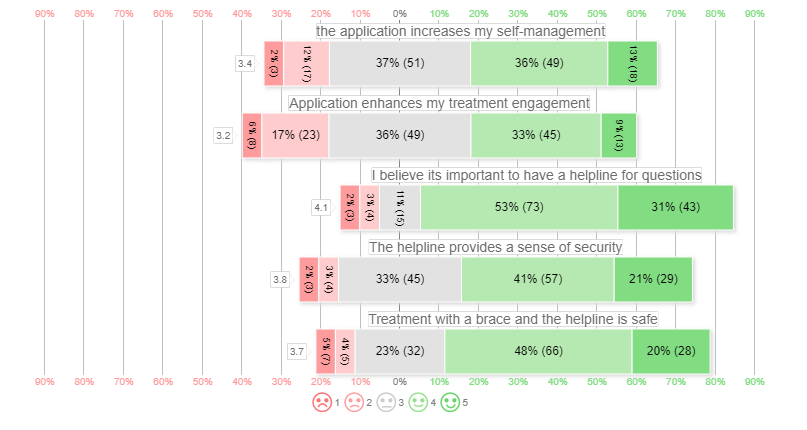
 5-point Likert scales with 1 and red indicating ‘ totally disagree’ to 5 and green ‘totally agree’

mean of Likert scale score
