# Supplementary material for: Patient Experiences With a Mobile Self-Care Solution for Low-Complex Orthopedic Injuries: Mixed Methods Study
Source: JMIR Hum Factors. 2025 Mar 14;12:e53074. doi: 10.2196/53074 (PMC11927796; doi:10.2196/53074)
Supplement: Multimedia Appendix 7 [file humanfactors-v12-e53074-s007.docx]

**Multimedia Appendix 7.** Frequency of limitation per week in physical function, activities of daily living, and school or work in patients treated with DD at three months follow-up

|  | **Activities of daily living** | **Sports activities** | **Limited in school or work** |
| --- | --- | --- | --- |
| Not (0 times per week) | 84 (61%) | 72 (52%) | 81 (59%) |
| Sometimes (1-2 times per week) | 28 (20%) | 35 (25%) | 39 (28%) |
| Often (3-5 times per week) | 15 (11%) | 17 (12%) | 17 (12%) |
| Very often (every day) | 11 (8%) | 14 (10%) | 1 (1%) |
